# Engaging the urban poor in community action on social determinants of health — lessons from the ‘*Mahila Arogya Samiti*’ model in the Indian state of Chhattisgarh

**DOI:** 10.1186/s44263-023-00034-1

**Published:** 2024-01-03

**Authors:** Samir Garg, Shriyuta Abhishek, Mukesh Dewangan, Ashu Sahu, Lalita Xalxo, Prabodh Nanda, Pradeep Tandan, Mohammad Jawed Quereishi, Anand Kumar Sahu

**Affiliations:** 1 State Health Resource Centre, Chhattisgarh, Raipur India; 2 State Programme Management Unit, National Health Mission, Chhattisgarh, Raipur India

The example of women’s health committees known as *Mahila Arogya Samiti* in Chhattisgarh stands out among government initiatives on community participation among urban poor in India. The initiative builds on the agency and solidarity of working-class women. The autonomy that these committees enjoy helps them to adopt a people-centred agenda.

## Background

From Alma Ata in 1978 to recent times, many declarations of the World Health Organisation and United Nations have emphasised the need for community participation in health. In these declarations, a comprehensive definition of health was adopted that included social determinants of health. In addition, community participation was understood as empowerment of people to play a central role in making decisions related to their own health. Countries had committed to creating the conditions for community participation to further the goals of human rights and participatory governance in health [1].

The actual progress in ensuring community participation has been far less than satisfactory in many low- and middle-income countries (LMICs) including India [2]. Though most health programmes have a component on community participation, they often define it in very narrow terms. Usually, the objective is limited to ensuring enough utilisation of the specific services offered by the programme [3]. Action on social determinants of health is rarely promoted by health systems as they often deem those to be the responsibility of other sectors such as education, food security, water and sanitation [4]. Implementers of health programmes, especially governments, often struggle to attract relevant communities when trying to promote committees or collectives for involvement of people. The urban poor in India, i.e. the socio-economically disadvantaged families and individuals mostly living in slums, face extreme inequity in access to basic services, including healthcare. Engaging the urban poor is considered to be further challenging due to the greater heterogeneity among this population [5].

The experience of *Mahila Arogya Samiti* (MAS) in the state of Chhattisgarh under India’s National Urban Health Mission (NUHM) can offer useful lessons in creating genuine community participation of the urban poor in health. MAS, initiated in Chhattisgarh in 2013, are community health committees focusing on action on the social determinants of health [6]. Each MAS consists of ten women belonging to a local slum community. Currently, there are 3700 active MAS committees in the urban slums of Chhattisgarh. MAS have demonstrated a consistent track record of acting on the social determinants of health including in domains such as community-level health services, food security and nutrition, gender-based violence, safe drinking water and sanitation. They have been able to use a range of strategies for their action — monitoring public services to identify problems in delivery, helping spread information on human rights, creating community consensus, negotiating with elected officials of local government, registering grievances with government authorities and organising large gatherings to raise issues and put pressure on government agencies to improve services. They have enjoyed an encouraging amount of success in getting many public health problems resolved, e.g. in delivery of food security entitlements and safe drinking water [7].

## What underlies the success of MAS?

Each MAS was formed through a participatory process and women played the main role in its functioning. Each MAS represented around 100–200 households: it consisted of ten women as members, and each member was selected by a set of 10–20 contiguous households to represent them. The ten members then selected two amongst them to be the officer bearers who chair the meetings of MAS and manage its finances. The state had a community health worker (CHW) programme promoted by the NUHM and it acted as a facilitator for MAS including in their formation. While the CHWs provided a significant amount of information and support to MAS, they did not act as leaders of MAS. The leadership of MAS remained in the hands of its office bearers. The MAS were provided with an annual round of training by the government, which was designed to reflect the stated objectives of the MAS — to monitor and act on social determinants of health [6]. The training provided the MAS with a human rights orientation and inculcated values of inclusiveness that oppose discrimination on grounds of caste, religion, gender and language. It also built capacities in terms of information on rights and entitlements people have [8]. A government-civil society collaboration provided the necessary capacity for implementing the training. The funds for training of MAS were allocated by government and the civil society partner contributed to the training content and execution. Mechanisms for interactions between MAS of different areas were promoted for sharing experiences, successes, challenges and lessons learnt among each other and to build bonds beyond the immediate neighbourhood. Another key platform was the annual *Jan Samwad Sammelan*, a public dialogue event that involved a large gathering of MAS committees in the municipality demanding accountability from government civil servants and elected officials [9].

## Agenda, accountability and recognition

The MAS were responsible for setting the agenda for their actions. They based it on the actual needs of people, often beyond what their training covered. For instance, they chose to work on improving the social environment and safety for children and women by opposing practices of gambling and substance abuse in common spaces. The government did not impose any set of tasks on MAS, and the MAS were only accountable to the urban poor populations whom they represented. The autonomy and the nature of the accountability relations built into the programme allowed the MAS to grow as an empowered entity and to act on a people-centred agenda.

The MAS provided women with an opportunity to participate in a larger social sphere, beyond their homes and other workplaces. It allowed them to interact with other women from underprivileged backgrounds and to build bonds of sisterhood. The women’s collective of MAS allowed them to build capacity, and it empowered them to act for the good of their fellow women and the urban poor, motivated by a sense of solidarity with them. Their efforts and successes earned them substantial social recognition from their neighbourhood communities, further motivating them to take greater risk and effort for getting bigger problems solved. The social recognition as women leaders in their communities and beyond also helped them to demand the attention of local politicians and officials.

## Conclusions

The MAS experience in Chhattisgarh offers valuable lessons for LMICs in terms of how governments can facilitate community participation by providing an enabling framework involving a participatory constitution of MAS with women leadership, effective arrangements for capacity building and appropriate accountability relationships. Yet, considerable scope remains to strengthen MAS further. Currently, MAS have to ensure that they are being heard and that the government acts in response to their complaints. There are no binding directives that mandate the government to act on the feedback from MAS on public programmes. In this regard, India can learn from the community engagement policies enacted by countries such as Brazil where institutional and legal provisions have been created to allow people’s health committees a seat at the table during government negotiations related to health services [10].

## Data Availability

Not applicable.
